# Metabarcoding study to reveal the structural community of strongylid nematodes in domesticated horses in Thailand

**DOI:** 10.1186/s12917-024-03934-y

**Published:** 2024-02-24

**Authors:** Mohamed H. Hamad, Sk Injamamul Islam, Wanarit Jitsamai, Teerapol Chinkangsadarn, Darm Naraporn, Suraseha Ouisuwan, Piyanan Taweethavonsawat

**Affiliations:** 1 The International Graduate Program of Veterinary Science and Technology (VST), Faculty of Veterinary Science, Chulalongkorn University, Bangkok, 10330 Thailand; 2 Infectious Diseases, Department of Animal Medicine, Faculty of Veterinary Medicine, Zagazig University, Zagazig, 44511 Egypt; 3 Parasitology Unit, Department of Veterinary Pathology, Faculty of Veterinary Science, Chulalongkorn University, Bangkok, 10330 Thailand; 4 Department of Parasitology and Entomology, Faculty of Public Health, Mahidol University, Bangkok, Thailand; 5 Department of Surgery, Faculty of Veterinary Science, Chulalongkorn University, Bangkok, 10330 Thailand; 6 Horse Farm and Laboratory Animal Breeding Center, Queen Saovabha Memorial Institute, The Thai Red Cross Society, Hua-Hin, Prachuap Khiri Khan Province 77110 Thailand; 7 Biomarkers in Animals Parasitology Research Unit, Chulalongkorn University, Bangkok, 10330 Thailand

**Keywords:** Deep amplicon sequencing, Horses, Nemabiome, Strongylid parasites

## Abstract

**Background:**

Mixed strongylid infections significantly impact equine health and performance. Traditional microscopy-based methods exhibit limitations in accurately identifying strongylid species. Nemabiome deep amplicon sequencing approach previously succeeded in describing the strongylid communities in livestock including equids. However, there are no available studies that describe the structural communities of strongylid parasites in horses in Thailand. Therefore, this study was undertaken encompassing the ITS-2 rDNA metabarcoding assay to characterize strongylid species within horse fecal samples collected from a cohort of yearlings at the largest domesticated stud farm in Thailand. In addition, to investigate the capability of ITS-2 rDNA in assessing the phylogenetic relationships among the identified strongylid species.

**Results:**

The study identified 14 strongylid species in the examined equine populations, each with varying prevalence. Notably, *Cylicocyclus nassatus* and *Cylicostephanus longibursatus* were identified as the predominant species, with *Strongylus* spp. conspicuously absent. The phylogenetic analysis of 207 amplicon sequence variants (ASVs) displayed a complex relationship among the investigated cyathostomin species, with some species are positioned across multiple clades, demonstrating close associations with various species and genera.

**Conclusion:**

The ITS-2 nemabiome sequencing technique provided a detailed picture of horse strongylid parasite species in the studied population. This establishes a foundation for future investigations into the resistance status of these parasites and enables efforts to mitigate their impact.

**Supplementary Information:**

The online version contains supplementary material available at 10.1186/s12917-024-03934-y.

## Background

Strongylids are a group of parasitic nematodes that infect horses and other equids worldwide, with a potential negative impact on equine health and performance [1, 2]. These nematodes have a complex life cycle, involving multiple stages of development within the host and on the pasture [3, 4]. Of these parasites, the Cyathostominae subfamily, commonly referred to as cyathostomins or small strongyles, has become the most significant group [2]. These parasites, primarily in their adult stage, cause damage to the intestinal lining, leading to anemia, weight loss, diarrhea, colic, and death in severe cases, especially in young or debilitated animals [5].

One of the challenges in studying strongylid nematodes is their high species diversity, with over 50 species known to infect horses [6]. Identification of different species is necessary to develop effective control strategies and understand the epidemiology of these parasites [7]. Traditional morphological methods to identify a wide range of strongylid parasites have limitations due to subtle differences in egg and larvae morphology among species, making it difficult to distinguish them accurately [8, 9]. In contrast, the emergence of modern molecular techniques has considerably enhanced non-invasive diagnostic methods [10].

Although molecular methods such as PCR-based assays (PCR-Reverse Line Blot (RLB) and real time qPCR) have been used for the identification of nematodes in horses, they also have some limitations, such as the need for species-specific primers and probes, potential cross-reactivity, and an inability to detect unknown or unexpected species [11]. Furthermore, proper optimization and validation of these assays are necessary for accurate quantification across laboratories [8, 12]. To overcome these limitations, metabarcoding has emerged as a promising alternative method to study nematode communities in different environments [7, 13, 14]. This method serves as a quick and noninvasive diagnostic tool, enabling researchers to investigate the variety of nematode species present and their distribution across different populations in various animals, including sheep [15], dairy heifers [16], equines [10, 17]. Additionally, it facilitates the exploration of dynamic changes over time within individual hosts and across populations [18, 19]. Metabarcoding allows pooling and extensive sequencing of hundreds of samples in a single run, reducing the cost per sample [20]. It generates a large amount of data without employing species-specific primers, allowing the identification of rare species that morphology and other approaches may miss [21], thus providing a comprehensive analysis of the parasite community present in each horse population.

Investigating strongylid infections and devising strategies to counter the escalating issue of parasitic resistance has become a worldwide concern. Numerous research studies have predominantly centered around examining strongylid infections in cattle [22, 23], goats [24–26], pigs [27], and dogs [28, 29] within the region of Thailand. However, there is a lack of available studies on strongylid parasites and their prevalence in horses.

The equine population in Thailand holds substantial importance across diverse sectors such as agriculture, racing, and tourism purposes. In addition, the Horse Farm and Laboratory Animal Breeding Center, operated by the Thai Red Cross Society in Phetchaburi, housing one of the country’s largest horse populations, this center carries out the crucial mission of producing antitoxin serum for snakebite treatment, a task demanding attention to equine health and parasite management practices. Thus, this study aimed to employ advanced ITS-2 rDNA metabarcoding technique to meticulously identify the diverse strongylid species in horses present at this farm. Additionally, to investigate the capability of ITS-2 rDNA in assessing the phylogenetic relationships among the identified strongylid species.

## Methods

### Samples collection, fecal examination, and larval culture

The study population consisted of two distinct sections (referred as A and B throughout the study) within the Horse Farm and Laboratory Animal Breeding Center, Thai Red Cross Society, in Phetchaburi, Thailand. Additional file 1: Figure S1 outlines the workflow of the sampling and examination process. All horses underwent deworming every six months as a routine healthcare practice. For the purposes of this study, it was confirmed that horses within the studied sections had not received any deworming treatment for the last six months. The separation of horses into different sections was not intentional for the study but a normal practice on the farm. The reason for this separation was that different groups of horses were used for toxin serum production. This separation was necessary to maintain their designated functions and prevent any potential cross-contamination between horse populations. Section A housed 57 horses, and second section B housed 48 horses. Horses in each section were allowed access to their own grazing pasture throughout the day. The age range of the included horses was between two and four years old, and they were provided with appropriate and balanced diets, along with regular grooming as part of their standard healthcare regimen.

A total of 105 horse fecal samples were submitted to the parasitology unit, faculty of Veterinary Science, Chulalongkorn University, Bangkok, Thailand between October and November 2022. The samples were collected rectally from individual horses in the two selected sections, without consideration of their age, sex, or breed. The samples were examined using the floatation technique for initial qualitative screening and the McMaster method for subsequent quantitative analysis of confirmed positive samples. The McMaster method employed a multiplication factor of 50 to account for the dilution ratio and accurately calculate eggs per gram of feces (EPG) [30]. Data was recorded on a spreadsheet (Microsoft Excel®) for subsequent data analysis (Additional file 2: Table S1). Samples with a strongylid egg count of 200 EPG or higher were included in the study, resulting in a total of 50 samples (25 from each section). An equal amount (40-gram fecal portion) from each horse was cultured for seven days at room temperature (∼ 25 °C) [31]. Afterward, L3 larvae were harvested from individual samples and then pooled for each section into separate tubes. Each tube contained aliquots of approximately 2000 larvae [32], followed by preservation in 70% ethanol for further analysis. This process resulted in a single pooled sample per section.

### DNA extraction, amplification, and sequencing

Genomic DNA for each sample was extracted from L3 larvae using a NucleoSpin® Tissue DNA extraction kit. The purity and concentration were checked using a NanoDrop™ Lite Spectrophotometer. Amplification and purification of ITS-2 amplicons have been performed following the protocol described in [32] and further details are available at https://www.nemabiome.ca/. In brief, two rounds of PCR were used to generate sequencing libraries of ITS-2 amplicon. In the initial PCR round, the ITS-2 rDNA fragment was amplified using NC1 and NC2 primers [33] incorporating with adapter sequences. The PCR reaction included 0.5 µl of KAPA HiFi Polymerase (KAPA Biosystems, UK), 0.75 µl of dNTPs (10mM), 5 µl KAPA HiFi Buffer (KAPA Biosystems), 0.75 µl each of forward and reverse primer mix (10 µM), 13.25 µl of molecular-grade water, and 1 µl of template DNA. Thermocycling conditions consisted of an initial denaturation at 95 °C for 2 min, followed by 35 cycles of 98 °C for 20 s for denaturation, 62 °C for 15 s for annealing, and 72 °C for 15 s for extension, concluding with a final extension at 72 °C for 2 min. The amplified PCR products were confirmed through gel electrophoresis on a 1.5% agarose gel. Subsequently, the PCR products underwent purification using magnetic bead technology (AMPure XP Magnetic Beads (1X), Beckman Coulter, Inc.).

The second PCR involved the addition of Illumina barcoded primers and utilized the first PCR product (2 µl) as the template. The reaction mixture included 5 µl KAPA HiFi Buffer (KAPA Biosystems), 1.25 µl each of forward and reverse primers (10 µM), 0.75 µl dNTPs (10 mM), 0.5 µl KAPA HiFi Polymerase, and 14.25 µl molecular-grade water. Cycling conditions comprised an initial denaturation at 98 °C for 45 s, followed by 7 cycles of 98 °C for 20 s for denaturation, 63 °C for 20 s for annealing, and 72 °C for 2 min for final extension. Amplicons were then purified using magnetic bead technology. The concentration of each purified amplicon was determined by using a Qubit Flex Fluorometer (Invitrogen) and TapeStation 4200 (Agilent). After quantification, the samples were pooled to form a single library (100 ng per sample). For sequencing, MiSeq Reagent Kit v2 (500 cycles) (Cat. MS-102-2003, Illumina), which adopts the sequencing-by-synthesis method, were used to generate paired-end reads. The raw sequencing data were deposited in the GenBank/SRA database under the BioProject accession number PRJNA997452.

### Bioinformatic analysis of amplicon sequences

The raw amplicon sequencing data were initially demultiplexed and the indices removed using standard post-run processing for the MiSeq platform. There were 156,930 and 163,653 demultiplexed reads for first and second population, respectively. The details regarding the total number of sequenced reads and the resulting reads after bioinformatic process are described in Additional file 2: Table S2. The data processing steps were carried out using DADA2 version 1.26.0 and its associated ITS-2 pipeline workflow [34], with specific adaptations made in accordance with the approach employed by Poissant et al. [10]. Specifically, reads containing ambiguous bases (Ns) were removed using the *filterAndTrim* function (maxN = 0). Primers were removed using Cutadapt version 4.0 with forward and reverse primers and their reverse complements as inputs [35]. The *filterAndTrim* function was then applied to discard reads with two maximum expected errors in the forward direction or five in the reverse direction, reads containing PhiX spike-ins, reads with bases having quality scores of two or less, and reads less than 200 bp long. An error model was constructed using the *learnErrors* command and error correction was conducted using the *dada* function with sample pooling set to TRUE. The corrected forward and reverse reads were combined using the *mergePairs* command, allowing up to 1.5% base mismatches in the overlapping region. The implementation of the maximum mismatch as a proportion of the overlap region was adopted instead of a fixed value. The resulting merged sequences were then clustered based on their sequence similarity using the *makesequence* command, which generated a table of 207 unique amplicon sequence variants (ASVs) and their frequencies. Chimeric sequences were removed using the *removeBimeraDenovo* command with the method = consensus parameter. The taxonomic assignment of unique sequences was performed using the *assignTaxonomy* command with an 80% bootstrap cutoff. This threshold is frequently used in molecular ecology due to its ability to balance accuracy and sensitivity in taxonomic classification, especially for high-throughput sequencing data [10, 36]. The ITS-2 taxonomy reference library was curated following Poissant et al. [10], the database was carefully edited to eliminate incomplete or potentially inaccurate sequences. The custom curated reference data is available in Additional file 3.

### Development of ITS-2 reference library

An initial taxonomic assignment was constructed by utilizing the nematode ITS-2 Database version 1.5.0 [37], which is accessible at www.nemabiome.ca/its2-database.html. This process revealed a total of 16 species of horse strongylid parasites. All available ITS-2 sequences belonging to these species were obtained from the nemabiome database, resulting in a total of 225 sequences. Next, a multiple sequence alignment (MSA) was performed using the *mafft* tool [38] and visualized in the CLC sequence viewer 8.0 [39]. Sequences found to be incomplete were removed. Subsequently, phylogenetic trees were constructed using the maximum likelihood approach with 1,000 bootstrap replicates in IQ-TREE [40] to elucidate potential errors in species identification based on unexpected clustering. Sequences showing evident misalignments were deemed likely to result from species misidentification and were subsequently excluded from the reference library [7, 10]. Details about excluded sequences are provided in (Additional file 1: Table S3). This refinement process was carried out four times to ensure accurate species segregation in the final phylogenetic tree (Additional file 1: Figure S2). After undergoing this refining procedure, a total of 197 sequences were retained for the reference library (Additional file 3).

### Phylogeny and relative abundance analysis of strongylid nematodes in field samples

The assigned sequences from the samples were aligned using the *mafft* tool [38] and visualized using the CLC sequence viewer 8.0 [39]. A maximum likelihood phylogenetic tree was constructed with 1,000 bootstrap replicates using IQ-TREE, incorporating 207 nucleotide sequences. The resulting tree was visualized and annotated using iTOL (Interactive Tree Of Life) [41]. Additionally, the relative abundance of each strongylid species was obtained by dividing the total reads assigned to each species by the total number of reads per sample. Species with a prevalence below 0.05% were excluded from the analysis, as such low abundance levels suggest the presence of less than one larvae per sample, likely attributable to contamination or erroneous read identification [7, 32].

### Fecal egg count data analysis

Data was processed using OriginPro 9.7 (OriginLab Corporation©, Northampton, MA, USA). The mean fecal egg counts (FECs) were calculated as the average of all recorded strongylid EPG, including zero values. Meanwhile, the mean intensity (MI) was determined as the average number of EPG for positive horses within each Sect. [42]. Normality of the data was assessed using Shapiro–Wilk and Kolmogorov–Smirnov tests, revealing a non-normal distribution. Mean intensity for the two sections was evaluated using the non-parametric Mann-Whitney test, with a critical probability set at *P* = 0.05.

## Results

### Community composition with relative abundance of strongylid nematode species

**Fig. 1:**
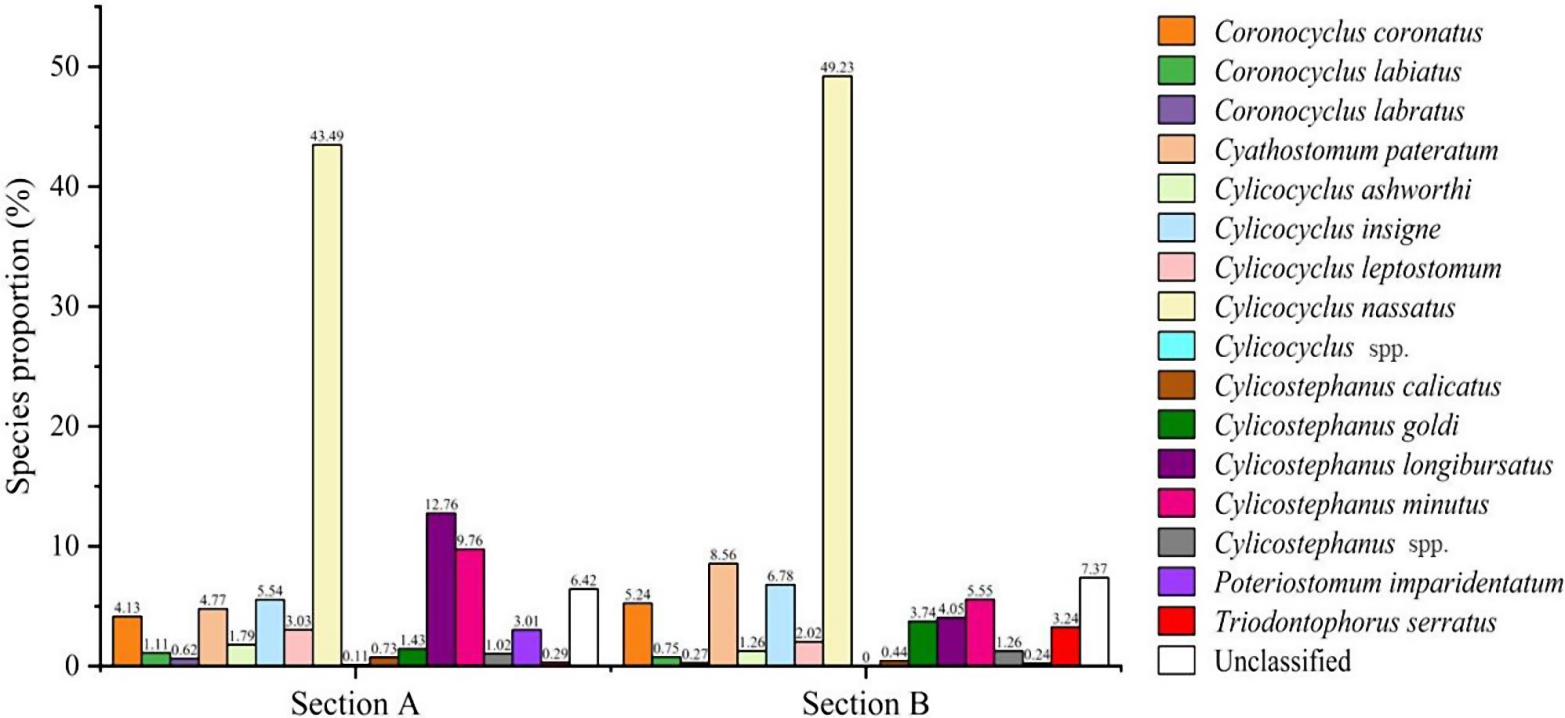
Species proportion (%) of strongylid nematodes based on amplicon sequence variants at taxa level from two separate pooled horse samples. Each taxon is represented by a distinct color

DADA2 analysis revealed a total of 207 ASVs within the two pooled fecal samples. Of these, 181 ASVs were assigned to the genus level, and 173 ASVs were confidently classified at the species level. However, 26 amplicon sequences could not be classified with a minimum bootstrap support of 80% (Additional file 2: Table S4). These ASVs were distributed into two distinct species communities, encompassing 152 ASVs in section A and 132 ASVs in section B. These ASVs collectively represented 14 strongylid species, each demonstrating distinct relative abundances within the investigated sections. Notably, no sequences belonging to *Strongylus* group were detected. *C. nassatus* prominently emerged as the most prevalent species in both communities, accounting for relative abundances of 43.49% and 49.23% in section A and section B, respectively. Conversely, less frequently encountered species were identified, with *T. serratus* exhibiting the lowest relative abundance (0.29%) in section A, while *P. imparidentatum* emerged as the least common species in section B with a relative abundance of 0.24% (Fig. 1). In addition, FEC analysis revealed that non-significant difference was found between the mean infection intensity rates for animals housed in sections A and B (Mann-Whitney test, *U* = 599.5; *p* = 0.796). The mean EPG and MI of the examined groups are provided in Table 1.

**Table 1:** Mean egg per gram (EPG) values and mean intensity (MI) of EPG reported for examined sections (A and B)

Section	Examined horses	Mean EPG ± SD	MI EPG ± SD	Horses with ≥ 200 EPG (%)
Section A	57	364.04 ± 532.16	610.29 ± 570.72	25 (59.65%)
Section B	48	385.42 ± 504.65	544.12 ± 523.21	25 (70.83%)

*AbbreviationEPG* Eggs per gram, *MI* Mean intensity, *SD* Standard deviation

### Phylogenetic analysis reveals the complex community of strongylid nematodes

**Fig. 2:**
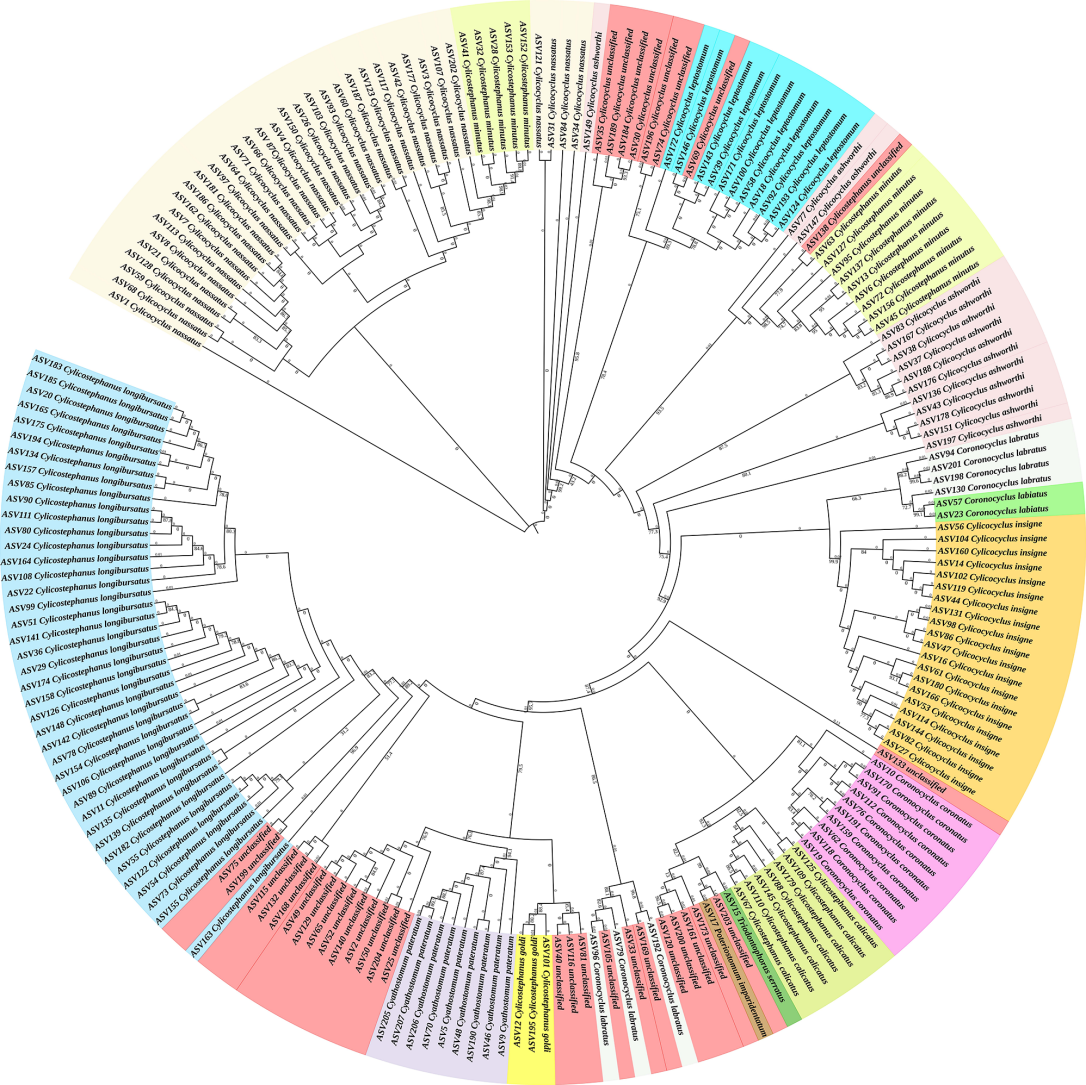
Maximum likelihood phylogenetic diversity of 207 sequences of strongylid nematodes in the equine population of Thailand, as elucidated through nemabiome metabarcoding analysis. The sequences are distinguished by color, representing their respective species. The numerical annotations at the branch origins denote bootstrap values, while those near the branch endpoints indicate branch lengths

The conducted phylogenetic analysis of 207 amplicon sequence variants (ASVs) based on ITS-2 DNA sequencing, using the maximum likelihood method (Fig. 2), revealed a highly intricate community of strongylid nematodes, including Cyathostominae and Strongylinae. The ASVs were found to be distributed across various branches, representing six distinct genera: *Cyathostomum*, *Cylicocyclus*, *Cylicostephanus*, *Coronocyclus*, *Poteriostomum*, and *Triodontophorus*, each with multiple ASVs. The separation between some species within the phylogenetic tree appears to be ambiguous and not completely distinct, as the sequences representing *C. minutus*, *C. ashworthi*, and *C. labratus* are present in two different clades. Furthermore, groups of sequences which could not be classified beyond Cyathostominae subfamily level are distributed across multiple clades throughout the tree. On the other hand, the unclassified *Cylicocyclus* spp. were found adjacent to *C. ashworthi* and *C. leptostomum*, from the same genera and unclassified *Cylicostephanus* sequence was found alongside *C. minutus*. The sequences representing certain species, including *C. pateratum*, *C. nassatus*, *C. insigne*, *C. longibursatus*, *C. calicatus*, *C. goldi*, *C. coronatus* and *C. labiatus* formed distinct monophyletic clades, some of which are closely related to each other.

## Discussion

Despite the evident clinical and health implications of strongylid parasites in horses, there has been limited research on the molecular identification and diversity of strongylid populations in different regions, including Thailand. Accurate identification of species is crucial to study their biology, epidemiology, and pathogenicity [43]. Our work used a nemabiome metabarcoding approach to identify strongylid nematodes within two distinct populations at the largest horse farm in Thailand. The decision was made to group the samples in pairs, considering that these two-horse populations are housed in two different sections and do not have access to the same pasture. This approach aims to account for potential variations in parasite diversity between the distinct sections.

In this study, the ITS-2 metabarcoding technique successfully identified 14 strongylid species, with *C. nassatus* and *C. longibursatus* presenting the highest relative abundance values. These findings support the suggestion that these species may account for the vast majority, approximately 98–99%, of the global cyathostomin burden [44–46]. Furthermore, our study identified one large non-migratory strongylids (*T. serratus*). This species is characterized by its large size and relatively high fecundity, which suggests that it may have a different life history strategy compared to other cyathostomin species [47]. While *T. serratus* has been previously reported in Asian countries such as Japan, China, and South Korea, its prevalence and distribution in other regions is not well known [48–51]. It is noteworthy that our findings lacked any evidence of *Strongylus* species including highly pathogenic *S. vulgaris*. This absence may be attributed to the fact that all horses on the farm underwent routine deworming every six months. Large strongyles have a more extended prepatent period compared to small strongyles [52, 53], and thus, the deworming regimen may have impacted the detection of these parasites in our samples [54, 55]. Although the separate living environments and pastures, our investigation reveals consistent species communities between the two groups, albeit with varying prevalence. In addition, FEC count analysis revealed that there is no significant difference in infection intensities between the two horse populations. The persistence of shared species and similar infection intensities can be attributed to parallel environmental conditions, management practices, and deworming regimens adopted by both groups. This underscores the significant role these factors play in shaping strongylid communities [11]. Additionally, differences in species abundance suggest potential ecological niche specialization within each group, indicating species’ adaptability to specific conditions [41]. However, as noted by Poissant et al. [35], relative abundances inferred from molecular data can also vary due to PCR efficiency, genetic variations, cell numbers, as well as the choice of bioinformatic algorithms, parameters, and databases. These complexities and challenges underscore the difficulties associated with quantifying and interpreting species abundances through molecular techniques, especially considering varying morphological identification capabilities.

In the current study, the majority of amplicon sequence reads were confidently assigned to individual species, underscoring sufficient genetic distinction among the species in the reference sequence library. In addition, the cyathostomin sequences assigned only to the genus level (specifically *Cylicocyclus* spp. and *Cylicostephanus* spp.) may include some valid horse cyathostomin species, but their ITS-2 sequence data are not available due to the lack of reference sequence available for these species in the nemabiome database and ultimately in GenBank [10, 56]. For example, Traversa et al. [57] demonstrated that twenty-two Cytochrome oxidase c subunit I (COI) sequence haplotypes (overall 10.8% rate of intraspecific nucleotide difference) were identified within *C. nassatus* using specimens from different hosts and geographic origin. In contrast, minimal variation (ranging from 0.0 to 0.6% differences) was observed in the ITS-2 sequences, highlighting the presence of cryptic species not only within *C. nassatus* but also other cyathostomin species. The limited number of sequences that could not be classified beyond the subfamily level (Cyathostominae) may be attributed to the low genetic variation within this subfamily, making it difficult to distinguish individual species using ITS-2 rDNA sequences alone. Consequently, these sequences remain confined to the subfamily level based on their overall sequence similarity [7]. In such cases, the integration of multiple genetic markers can enhance phylogenetic understanding and aid in species identification. Combining ribosomal and mitochondrial haplotypes can provide complementary information, as the two markers have different rates of evolution and offer insights into different aspects of the species’ evolutionary history [7].

Despite employing molecular analysis, clear evidence supporting the distinct separation of cyathostomin species into their classical genera (i.e., *Cyathostomum*, *Cylicocyclus*, *Coronocyclus*, and *Cylicostephanus*) was not obtained. This discrepancy between the molecular and morphological classifications in the phylogram suggests that certain sequences in the ITS-2 database may have been inaccurately assigned due to difficulties in distinguishing indistinct or overlapping morphological traits [58]. The phylogenetic analysis of sequences representing nemabiomes in the study group of horses led to a complex and perplexing pattern. Some species appeared in multiple clades closely aligned with different species and genera. For example, *C. minutus* was found in two separate clades which may indicate the possibility of cryptic speciation, a phenomenon previously described in other studies [7, 59, 60]. Furthermore, the presence of two distinct groups formed by *C. ashworthi* and *C. labratus* may result from limited genetic differentiation among closely related species, making clear taxonomic assignments difficult [10]. This difficulty is compounded by complexities associated with the ITS-2 region, known for harboring paralogous sequences within a single organism [61]. The phylogenetic relatedness of unclassified *Cylicocyclus* spp. with *C. leptostomum* and *C. ashworthi*, as well as high levels of intraspecific diversity support the existence of cryptic species complexes, consistent with previous studies [7, 60]. The close phylogenetic relationship and genetic distance between a few sequences unable to be classified beyond the Cyathostominae subfamily level and *C. longibursatus*, *C. pateratum*, and *C. labratus* might indicate the presence of hybrids. This finding aligns with the previous study conducted by Sargison et al. [7]. The close phylogeny relationship among cyathostomin species could potentially enable interbreeding and hybridization, facilitating the transfer of adaptive genetic traits among them. Furthermore, the emergence of resistance to commonly used anthelmintic drugs is already a significant concern for multiple species [62] Thus, a deeper understanding of the phylogenetic relatedness of co-infecting cyathostomin species allows for the development of more precise deworming strategies. Through considering genetic similarities and differences, targeted treatments can be formulated, reducing the likelihood of resistance, and enhancing overall parasite management in equine health [7, 63].

### Limitations and future research

The limitation of this study arises from the use of pooled fecal samples. While this approach offers a broad overview, pooling may obscure individual-level variations in strongyle species composition. This could be addressed in future studies by supplementing pooled samples with individual analyses to capture finer details of the equine strongyle population. Additionally, the focus on a single stud farm may limit the generalizability of the study’s findings to broader equine populations in diverse geographical areas. This work also illustrates the power and limitations of ITS-2 nemabiome metabarcoding in equine studies. It enables the detection of a wide range of strongylid species, often identifying them at the species level. However, in some cases, sequences could only be identified at the genus level, emphasizing the need to continue to enrich DNA sequence databases for more comprehensive identification. Furthermore, the potential for paralogous ITS-2 copies within a genome can lead to inaccurate phylogenetic reconstructions. To overcome this, future research might consider complementary molecular markers to enhance specificity in species identification.

## Conclusions

In conclusion, this study utilized ITS-2 DNA metabarcoding techniques to identify strongyle species in equine fecal samples, offering a more comprehensive understanding than traditional approaches. The construction of a phylogenetic tree elucidated the evolutionary relationships among these parasites. These findings contribute to addressing a previously unexplored area of molecular evidence in Thailand, emphasizing the crucial role of molecular tools in advancing our comprehension of parasite taxonomy and evolution.

### Electronic supplementary material

Below is the link to the electronic supplementary material.

Supplementary Material 1

Supplementary Material 2

Supplementary Material 3

Supplementary Material 4

## Data Availability

All data generated or analyzed during this study are included in this published article. All the nucleotide sequences generated from this study have been deposited and are available in the GenBank database under the BioProject accession number PRJNA997452.
