# Supplementary figures and images for: Metabarcoding study to reveal the structural community of strongylid nematodes in domesticated horses in Thailand
Source: BMC Vet Res. 2024 Feb 24;20:70. doi: 10.1186/s12917-024-03934-y (PMC10893705; doi:10.1186/s12917-024-03934-y)

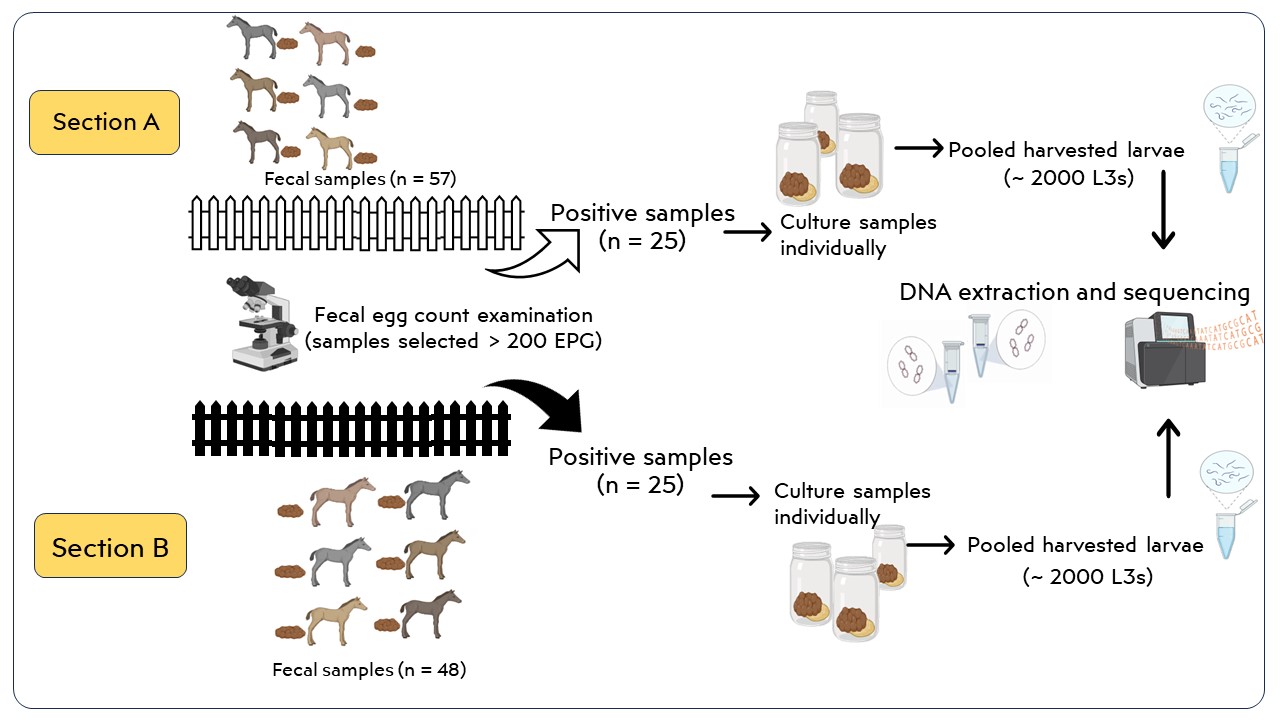


**Additional file 1: Figure S1**


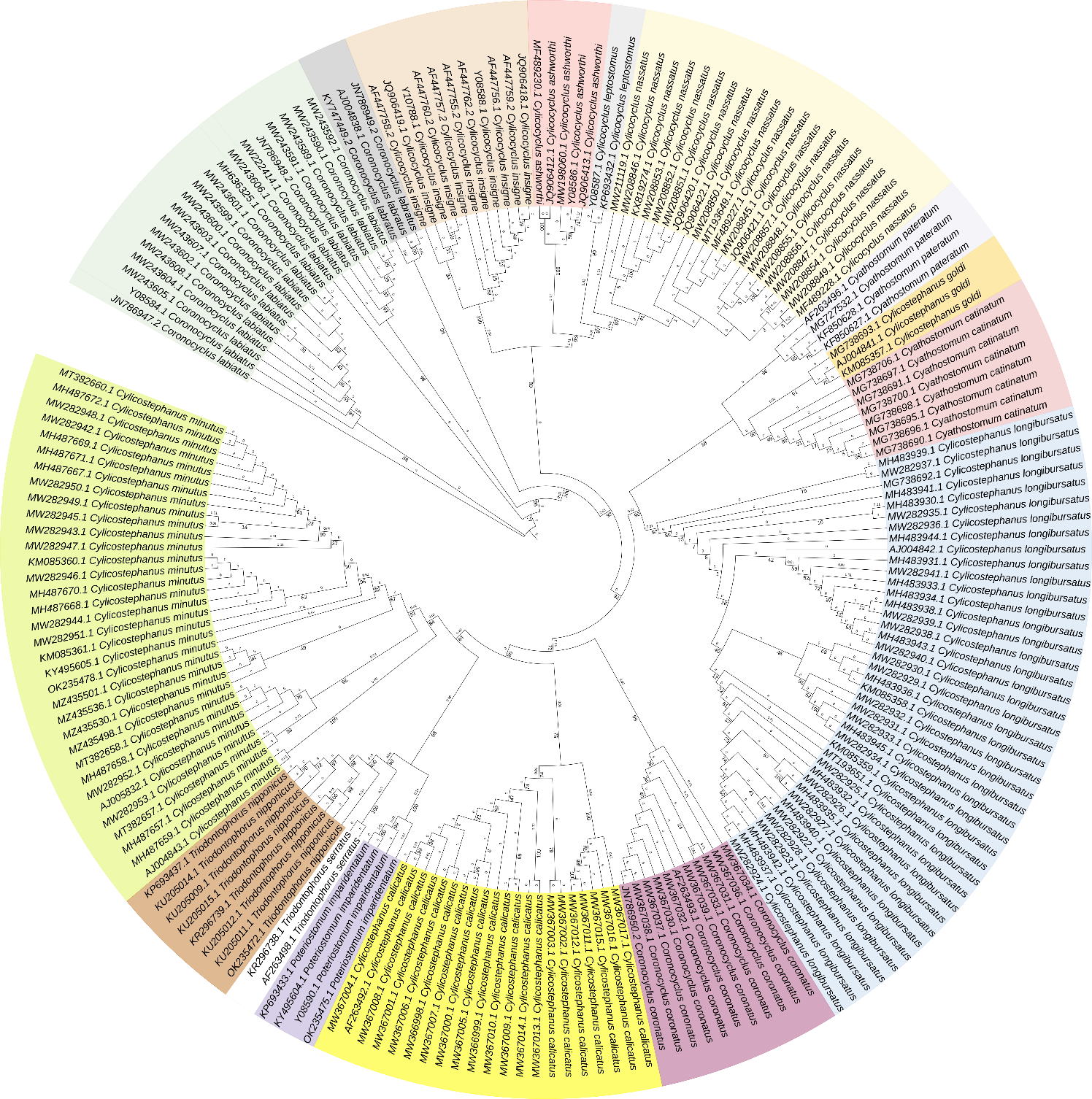


**Additional file 1: Figure S2**

Supplement: Supplementary file 2 — Supplementary Material 2 [file 12917_2024_3934_MOESM2_ESM.docx]
