# Supplementary material for: Metabarcoding study to reveal the structural community of strongylid nematodes in domesticated horses in Thailand
Source: BMC Vet Res. 2024 Feb 24;20:70. doi: 10.1186/s12917-024-03934-y (PMC10893705; doi:10.1186/s12917-024-03934-y)
Supplement: Supplementary file 4 — Supplementary Material 4 [file 12917_2024_3934_MOESM4_ESM.docx]

**Additional files**

Additional file [1](https://bmcvetres.biomedcentral.com/articles/10.1186/s12917-019-1929-2#MOESM1): Figure S1: The workflow of the sampling and examination process

Additional file [1](https://bmcvetres.biomedcentral.com/articles/10.1186/s12917-019-1929-2#MOESM1): Figure S2: A phylogenetic tree of the 197 horse strongyle ITS2 sequences included in nemabiome analyses

Additional file [2](https://bmcvetres.biomedcentral.com/articles/10.1186/s12917-019-1929-2#MOESM1): Table S1: Table S1 Raw data of individual horses examined, including Fecal Egg Counts (FECs)

Additional file [2](https://bmcvetres.biomedcentral.com/articles/10.1186/s12917-019-1929-2#MOESM1): Table S2 The number of sequenced and processed reads through the metabarcoding bioinformatics pipeline.

Additional file [2](https://bmcvetres.biomedcentral.com/articles/10.1186/s12917-019-1929-2#MOESM1): Table S3 Twenty-eight ITS-2 sequences excluded during the curation process of reference library

Additional file [2](https://bmcvetres.biomedcentral.com/articles/10.1186/s12917-019-1929-2#MOESM1): Table S4 Table of ASVs and Taxonomic Assignments for Strongylid Nematode Species isolated from Horses in Thailand

Additional file [3](https://bmcvetres.biomedcentral.com/articles/10.1186/s12917-019-1929-2#MOESM1): Taxonomy reference library
